# A dataset of conformer-independent structural molecular property indices for 33,715 organic molecules

**DOI:** 10.1016/j.dib.2026.113342

**Published:** 2026-10-09

**Authors:** Alexandra Farcaș, Lorentz Jäntschi

**Affiliations:** a Department of Physics and Chemistry, Technical University of Cluj-Napoca, European University of Technology (eUT+), Cluj-Napoca, 400641, Romania; b Department of Isotopic Technologies, National Institute for Research and Development of Isotopic and Molecular Technologies, Cluj-Napoca, 400293, Romania

**Keywords:** Molecular descriptors, SMPI, SMILES, Molecular graphs, QSAR/QSPR, Chemoinformatics, Ma- chine learning

## Abstract

This data article describes an openly accessible dataset hosted on Zenodo (DOI: 10.5281/zenodo.22974542), com- prising canonical SMILES representations and 121 struc- tural molecular property indices (SMPI) for 33,715 or- ganic molecules. Derived from molecular graph con- nectivity and Szeged-type vertex partitioning, the de- scriptors combine fragment-level atomic summaries, dis- tance weightings, interaction functions, matrix operators, and logarithmic transformations. Derived as a curated, quality-controlled benchmark subset from an initial 1008 theoretical operator pool, the resulting tab-separated file (33,715 rows, 122 columns) serves as a robust benchmark resource for QSAR/QSPR modeling, feature selection, chemical-space mapping, and machine-learning workflows requiring reproducible two-dimensional molecular repre- sentations.

Specifications Table

**Table:** 

Subject area Specific subject area	Chemistry; chemoinformatics; computational molecular science Conformer-independent graph- based molecular descriptors for QSAR/QSPR and machine learning
Type of data	Tab-separated UTF-8 text; canonical SMILES strings; numerical descrip- tor matrix
Data collection	Molecular structures were harmonized from structure files, explicit hydrogen atoms were included, and each molecule was represented as a connectivity graph. SMPI values were generated by a five-level oper- ator pipeline based on Szeged graph fragments and subjected to a 4-stage quality-control filter.
Data source location Data accessibility	Derived from a benchmark small- molecule library dataset DOI: https://doi.org/10.5281/zenodo.23121878; files: smpi-nog33715mols2.txt (filtered dataset) and filter121smpi.py (filtering script)
Related research article	A. Farcaș, L. Jäntschi, A molecu- lar descriptor dataset of 33,715 small molecules based on operator-based descriptor framework, Sci. Data 13 (2026) 786. DOI: https://doi.org/10.1038/s41597–026–07863–3

## 1 Value of the Data

• The data provide a fixed, reusable numerical rep- resentation of 33,715 organic structures without requiring three-dimensional coordinate generation, conformational sampling, or energy minimization.
• The 124 SMPI variables encode complementary combinations of molecular-graph fragmentation, atomic attributes, distances, interactions, aggregation statistics, and nonlinear transformations.
• The matrix can support QSAR/QSPR development, unsupervised chemical-space exploration, descriptor selection experiments, redundancy analysis, and comparisons of classical machine-learning algorithms.
• Canonical SMILES and descriptor values are stored together, enabling users to associate each numerical row with a machine-readable molecular structure and to derive additional representations when licensing and software conditions permit.
• The tab-separated plain-text format can be read directly by common scientific environments, including R, Python, Julia, MATLAB, and spreadsheet or database tools capable of handling wide tables.
• The dataset is useful for researchers studying the distributional properties of topological descriptors and the trade-off between inexpensive two-dimensional representations and more computationally intensive three-dimensional molecular features.
• An open-source Python generator script (smpi2026nog.py) is provided to compute the identical 124 SMPI descriptors for arbitrary new chemical structures directly from SMILES strings or SD files.
• Unlike uncurated descriptor sets, this dataset provides a pre-screened, non-redundant benchmark matrix derived by explicit variance, skewness, and collinearity (*|r| <* 0*.*95) filtering steps, enabling immediate ingestion into predictive statistical work- flows.

## 2 Background

Molecular descriptors translate chemical structures into numerical variables suitable for similarity analysis and statistical learning [1]. Classical two-dimensional in- dices are inexpensive and reproducible, but single-index families can be insensitive to changes in local atomic properties or bonding environments [2]. Conversely, three-dimensional representations may require conformer generation and can vary with geometry-selection proce- dures [3].

The structural molecular property indices (SMPI) ap- proach addresses this gap by combining graph partition- ing with a modular sequence of property, distance, inter- action, aggregation, and transformation operators [4,5]. Szeged-type fragmentations provide bond- or vertex- relative subdomains of a molecular graph and thereby permit local and global connectivity information to be encoded without relying on Cartesian coordinates [6,7]. Furthermore, integrating multi-property weightings (such as electronegativity, polarizability, and atomic mass) allows the descriptor space to capture electronic and topological features simultaneously, enhancing predic- tive power in quantitative structure-property relation- ships [8].

While our companion study in *Scientific Data* [5] de- posited the uncurated theoretical space of 1008 oper- ator algorithms to document the overarching genera- tion engine, the present data article describes a curated, non-redundant benchmark dataset specifically filtered for QSAR/QSPR applications. This data article provides a self-contained description of the deposited research ob- ject: its organization, algorithmic generation, multi-stage statistical quality controls, and reuse conditions. All op- erator definitions, filtering criteria, and workflow stages are fully detailed herein to enable independent ingestion and methodological application without reliance on ex- ternal manuscript iterations.

## 3 Data Description

The repository contains the file smpinog33715mols2.txt along with the genera- tor script smpi2026nog.py. It is a UTF-8 tab-separated text table with one header row and 33,715 data rows. The table has 125 columns: the first column stores a canonical SMILES representation, and the remaining 124 columns store SMPI variables. The logical organization of the deposited table is summarized in Table 1. Thus, the numerical portion can be represented as a matrix

(1) $$X\in R^{33715\times 124},$$

subject to the finite-value and domain restrictions in- troduced by the corresponding descriptor transforma- tions. This 33*,* 715 *×* 124 matrix represents the quality- controlled, non-redundant core selected from the broader 1008 raw operator space described in [5].

**Table 1:** Logical organization of the deposited table.

Component	Count	Description
Header	1 row	Field names separated by tab characters
Molecules Structure field	33,715 rows 1 column	One molecular record per row Canonical SMILES string
Descriptor fields	124 columns	Numerical SMPI values generated from molecular topology
Total fields	125 columns	Structure plus descriptor variables

Descriptor labels are compact operator codes. They should be treated as algorithm identifiers rather than as conventional chemical abbreviations. A code records the choices made along the construction pipeline. In gen- eral, the code positions denote the terminal transforma- tion and aggregation choices together with the interac- tion, distance, and atomic-property components. Exam- ples present in the dataset include IFEUB, IMETC, RIETA, LJEUD, IMEUF, and RNEUD. Because groups of descriptors can share one or more operators, users should not as- sume statistical independence solely from different col- umn names.

The file is analysis-ready in the sense that one row corresponds to one molecular structure and one descrip- tor column has a consistent algorithmic definition across all rows. Nevertheless, users should perform task-specific checks for duplicated structures, missing or non-finite val- ues, near-zero variance, scaling, and correlated variables before model fitting. Training/test separation should be carried out by molecule, preferably with scaffold-aware or other chemically informed splitting when the objective is prospective predictive assessment [9].

## 4 Experimental Design, Materials and Methods

The overall dataset-generation procedure is summarized in Fig. 1. The workflow begins with the harmonization of the molecular structures and the addition of explicit hydrogen atoms. Each structure is then represented as a molecular connectivity graph, thereby avoiding the need for three-dimensional geometry optimization or confor- mational sampling.

**Fig. 1:**
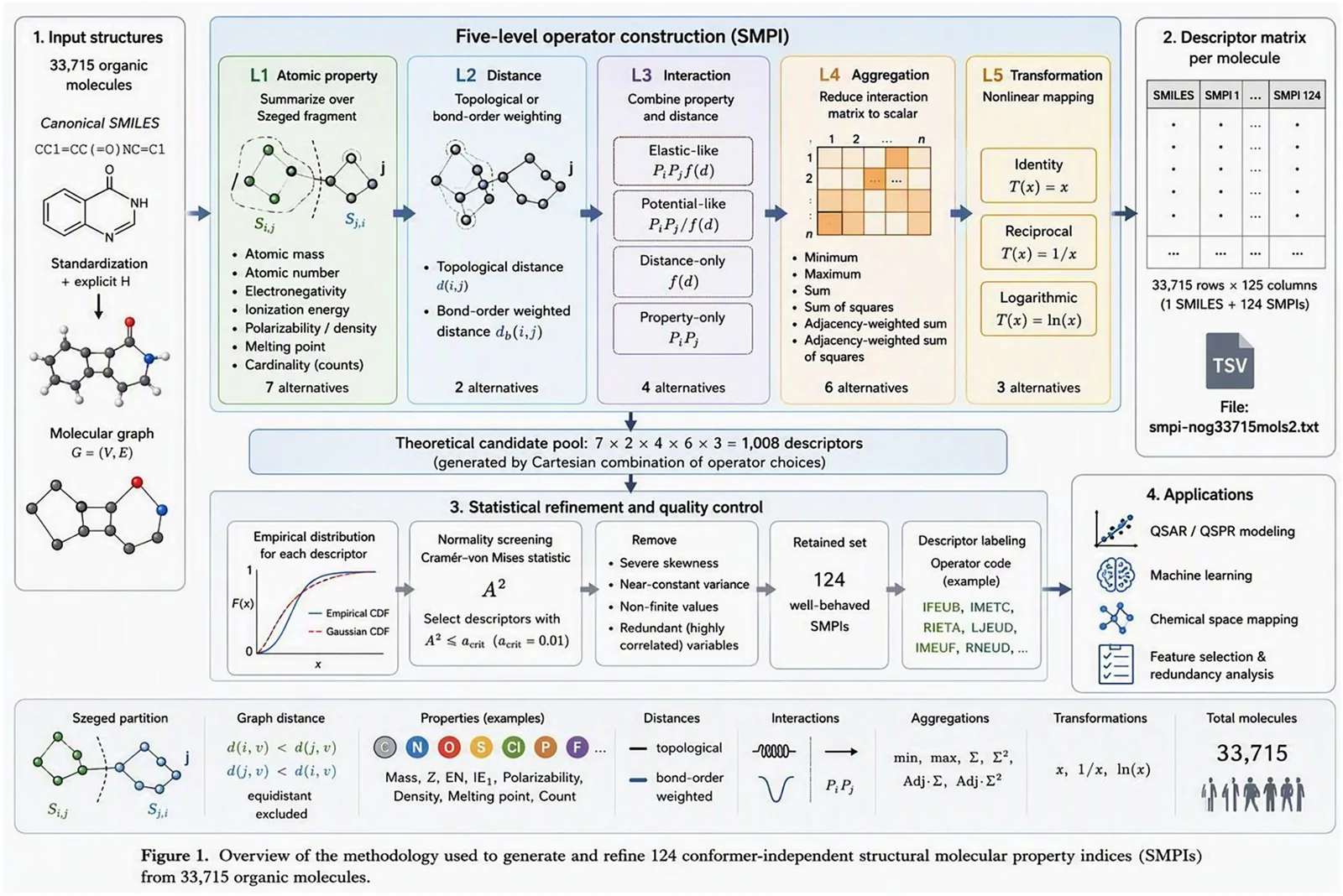
Overview of the workflow used to generate the conformer-independent SMPI dataset. Molecular structures are converted into connectivity graphs after structural harmonization and explicit-hydrogen addition. Szeged-type vertex partitioning is subsequently applied to define local graph fragments. A five-level operator pipeline combines fragment-based atomic properties, topological or bond-order-weighted distances, interaction functions, matrix aggre- gation operators, and final mathematical transformations. The resulting descriptor values are subjected to numerical and distributional quality controls before being assembled with the corresponding canonical SMILES representations in the deposited tab-separated dataset.

Szeged-type partitioning defines vertex-relative graph fragments that characterize the local topological environ- ments associated with pairs of atoms.

The resulting fragments are processed through the five- level SMPI operator hierarchy. The first level summarizes selected atomic properties within each fragment, whereas the second level defines the distance relation between the relevant vertices. The third level combines fragment properties and distances into pairwise interaction terms. Matrix-level aggregation is performed at the fourth level, and the fifth level applies the final identity, reciprocal, or logarithmic transformation. After numerical and dis- tributional quality control, the selected descriptor values are combined with the corresponding canonical SMILES strings to produce the publicly deposited data table.

### 4.1 Structural preprocessing

The source structures were processed in a common struc- tural format (such as canonical SMILES), and explicit hydrogen atoms were added prior to descriptor calcu- lation to ensure structural consistency. The structures were subsequently used as molecular connectivity graphs rather than as optimized three-dimensional conform- ers, preventing variability introduced by alternative con- former selection across the large library of 33,715 organic molecules [10]. Let a molecule be represented by a con- nected graph *G* = (*V, E*), where vertices correspond to atoms and edges correspond to chemical bonds. This representation makes the deposited variables invariant to rigid translation and rotation and avoids variability produced exclusively by selecting among alternative con- formers.

### 4.2 Szeged-type graph fragmentation

For vertices *i, j ∈ V* and graph distance *d*(*·, ·*), the frag- ment associated with *i* relative to *j* is

(2) Si,j={v∈V:d(v,i)<d(v,j)}.

The complementary fragment *S_j,i_* is defined analogously. Vertices equidistant from *i* and *j* do not belong to either strict fragment. This partitioning converts the relation between a selected pair of vertices into two graph domains whose sizes and property-weighted compositions can be summarized numerically [6]. Furthermore, fragmental graph decomposition enables capturing local topological environments that correlate strongly with macroscopic physicochemical properties.

### 4.3 Five-level operator construction

Candidate descriptors were generated using a hierarchi- cal operator sequence established within the Molecular Descriptors Family (MDF) methodology [7] (Table 2). At the first level, an atomic property or a vertex count was summarized over a Szeged fragment. The prop‑ erty set included atomic mass, atomic number, cardi- nality, solid-state density, electronegativity, first ioniza- tion energy, and melting point, using the aggregation rule assigned to each property in the generating algo- rithm. The second level introduced either a conven- tional topological path distance or a bond-order-weighted distance. The third level combined fragment prop‑ erties and distances through elastic-like, potential-like, distance-only, or property-only interactions. The fourth level reduced the pairwise interaction matrix by opera- tors such as a minimum, maximum, total sum, sum of squares, adjacency-weighted sum, or adjacency-weighted sum of squares. Finally, identity, reciprocal, or natural- logarithm functions were used to produce the reported scalar descriptor.

**Table 2:** Descriptor-generation hierarchy.

Level	Operator family	Function
L1	Atomic property	Summarizes a property over a graph fragment
L2	Distance	Defines topological or bond-order-weighted separation
L3	Interaction	Combines property and distance terms
L4	Aggregation	Reduces the pairwise matrix to a scalar
L5	Transformation	Applies identity, reciprocal, or logarithmic mapping

The Cartesian combination of the operator alternatives generated an initial theoretical family of

(3) 7×2×4×6×3=1008

candidate algorithms per molecule [5]. Descriptor calcu- lation was implemented in a standalone FreePascal pro-gram and applied independently to the molecular records [10]. The public data file contains the 124 descriptor columns selected for deposition together with the corre- sponding canonical SMILES strings [10].

### 4.4 Statistical refinement and quality control

To derive the final 124-descriptor benchmark matrix from the initial theoretical pool of *N*_0_ = 1008 candidate al- gorithms, a sequential four-stage filtering protocol was executed:

1. **Stage 1: Mathematical Domain and Finite- Value Check (***N*_1_ = 948 **retained).** Candidate al- gorithms were evaluated across all 33,715 molecular records for domain errors, division-by-zero, or nega- tive logarithm arguments. Any descriptor producing non-finite values (*±∞*, NaN) or zero-denominator er- rors was eliminated.
2. **Stage 2: Low-Variance and Constancy Filter- ing (***N*_2_ = 812 **retained).** Descriptors exhibiting zero variance (constant values) or near-zero variance (Var(*X*) *<* 10*^−^*^4^) across the dataset were removed to eliminate uninformative feature columns.
3. **Stage 3: Distributional Screening and Ex- treme Skewness Check (***N*_3_ = 426 **retained).** The empirical cumulative distribution function (eCDF) of each variable was compared against a nor- mal distribution parameterized by sample moments. Variables presenting extreme skewness (*|S| >* 5*.*0) or degenerate point-mass distributions were removed.
4. **Stage 4: Redundancy and Collinearity Filter- ing (***N*_4_ = 124 **retained).** Pairwise Pearson correlation coefficients (*r*) were computed across all re- maining variables. For clusters of descriptors dis- playing strong pairwise association (*|r| >* 0*.*95), redundant variables were pruned, retaining a sin- gle representative algorithm per operator sub-family with superior numerical stability and distributional balance.

The detailed breakdown and descriptor count after each sequential filtering stage are summarized in Table 3.

**Table 3:** Sequential descriptor filtering and quality- control pipeline.

Stage	Filter Type	Selection	Criteria & Thresholds
—	Initial Pool	Cartesian 7 *×* 2 *×* 4 *×* 6 *×* 3 operator space	1008
1	Domain Validity	Exclusion of non-finite values (NaN*, ±∞*)	948
2	Variance Filter	Exclusion of near-zero variance (Var(*X*) < 10*^−^*^4^)	812
3	Distributional	Exclusion of severe skewness (|*S| >* 5*.*0)	426
4	Redundancy	Collinearity pruning (*|r|≤*0*.*95 per sub- family)	**124**

At file-ingestion stage, four checks are recommended:

(i) verify the expected dimensions of 33,715 rows and 125 columns [10], (ii) preserve the SMILES column as text, (iii) convert only descriptor fields to numeric type, and (iv) identify missing, infinite, constant, and duplicate columns before scaling. For reproducible model compar- isons, all learned preprocessing operations, including im- putation, variance filtering, feature selection, and scaling, must be fitted only on the training partition.

### 4.5 Minimal reuse example

The following procedure reads the file without changing descriptor precision:

**Table:** 

Users should cite both this data article and the archived dataset when publishing analyses based on the deposited matrix [10].

### 4.6 Python filtering script

To ensure full computational reproducibility and trans- parency of the 4-stage quality-control and selection proce- dure, an open-source Python script (filter121smpi.py) was developed. The script automatically retrieves the raw 1008-descriptor archive from its companion Zenodo record (https://doi.org/10.5281/zenodo.21103707), executes constant, duplicate, collinearity, and statistical filtering stages, and extracts the final robust set of 121 descriptors. The standalone script is deposited in the Zenodo repository (https://doi.org/10.5281/zenodo.23121878). It can be executed via the command line as follows:

**Table:** 

### 4.7 Proof-of-concept QSPR benchmark workflow

To demonstrate the downstream utility of the SMPI ma- trix in QSAR/QSPR modeling without performing a full secondary research study, a proof-of-concept regression workflow was executed using the public ESOL aque- ous solubility benchmark dataset (log *S*, 1127 matched molecules) [11]. Molecular structures were matched with the SMPI dataset, and a baseline Random Forest regres- sor (100 estimators) was evaluated using 5-fold cross- validation across the 355 descriptor columns. The model achieved a robust cross-validated coefficient of determi- nation *R*^2^ = 0*.*858 *±* 0*.*031 (RMSE = 0*.*782 *±* 0*.*088), confirming that the 2D graph-based SMPI features en- code relevant physicochemical properties for quantitative regression tasks.

**Table:** 

### 4.8 Chemical space coverage

To evaluate the global distribution and structural dis- persion of the 33,715 organic molecules within the de- scriptor space, Principal Component Analysis (PCA) was performed on the z-score standardized SMPI feature ma- trix. The first three principal components account for 81.5% of the cumulative structural and topological vari- ance (PC_1_ = 43*.*8%, PC_2_ = 19*.*3%, and PC_3_ = 18*.*4%).

PC_1_ predominantly reflects molecular size and graph or- der, whereas PC_2_ and PC_3_ capture variations in vertex branching, fragment electronegativity weightings, and distance distributions across the molecular graph. This variance breakdown confirms that the 124 SMPI vari- ables span a continuous, well-dispersed region of two- dimensional chemical space suitable for diverse chemoin- formatics applications.

## Limitations

The dataset contains graph-derived topological descrip- tors and does not explicitly encode three-dimensional conformations, stereochemical configurations, conforma- tional flexibility, solvent effects, protonation-state en- sembles, tautomer distributions, or quantum-mechanical electronic structures [12,13]. While two-dimensional molecular representations provide high computational ef- ficiency and structural reproducibility across large chem- ical libraries, capturing spatial recognition and stereospe- cific interactions frequently requires complementary geo- metric or conformational features [13].

Furthermore, the repository provides independent molecular descriptors rather than measured biological or physicochemical endpoint labels; thus, supervised quan- titative structure-activity or structure-property relation- ship (QSAR/QSPR) applications require an indepen- dently curated response variable and robust validation procedures across diverse external test sets [14]. The source chemical library may not uniformly represent all regions of vast chemical space, meaning applicability- domain assessment remains essential prior to predictive deployment. Finally, univariate descriptor filtering or distributional screening does not guarantee low pair- wise multicollinearity or optimal predictive performance across every distinct endpoint, necessitating rigorous fea- ture selection protocols [14].

## Ethics Statement

The dataset consists of computed molecular-structure de- scriptors and contains no human participants, animal ex- periments, personal data, or social-media data. Ethical approval and informed consent were therefore not appli- cable.

## CRediT Author Statement

**Alexandra Farca¸s:** Data curation, Investigation, For- mal analysis, Writing – original draft. **Lorentz Jäntschi:** Conceptualization, Methodology, Software, Supervision, Validation, Writing – review and editing.

## Data Availability

The dataset and filtering workflow *SMPI-nog Filtered Descriptors and Selection Pipeline for 33,715 Organic Molecules* are openly available in Zenodo at https://doi.org/10.5281/zenodo.23121878. The deposited file- name is smpi-nog33715mols2.txt, accompanied by the deterministic Python filtering script filter121smpi.py.

## Funding

Publication of this work was supported by the Technical University of Cluj-Napoca, Romania, request 30116 of 28.09.2026.
